# Cognitive function and uric acid levels among low-income rural Chinese aged ≥60 years without hyperuricemia

**DOI:** 10.3389/fmed.2025.1753357

**Published:** 2026-01-14

**Authors:** Dongwang Qi, Juan Hao, Chanhong Shi, Jinli Zhou, Xuewei Yang, Hongwei Yue, Yanjia Wang, Jun Tu, Xianjia Ning, Zilong Chen

**Affiliations:** 1 Department of Neurology, Yiwu Central Hospital, The Affiliated Yiwu Hospital of Wenzhou Medical College, Zhejiang, China; 2 Department of Neurology, Tianjin Medical University General Hospital, Tianjin, China; 3 Department of Emergency Medicine, Yiwu Central Hospital, The Affiliated Yiwu Hospital of Wenzhou Medical College, Zhejiang, China; 4 Laboratory of Epidemiology, Tianjin Neurological Institute, Tianjin, China; 5 Tianjin Neurological Institute, Key Laboratory of Post-Neuroinjury Neuro-repair and Regeneration in Central Nervous System, Ministry of Education and Tianjin City, Tianjin, China

**Keywords:** cognitive impairment, elderly, epidemiology, rural population, uric acid

## Abstract

**Background:**

This study aimed to investigate the association between uric acid (UA) levels and cognitive function in a low-income, rural population in Chinese adults aged ≥60 years without hyperuricemia.

**Methods:**

Elderly individuals (≥60 years old) without hyperuricemia from rural areas of Tianjin were included in this cross-sectional study. Basic demographic and clinical information were collected, and cognitive impairment was assessed using the Mini-Mental State Examination (MMSE). Binary logistic regression analysis was performed to evaluate the association between UA levels and cognitive impairment, and multivariate linear regression analysis was used to explore the relationship between UA levels and MMSE scores. Subgroup analyses were conducted based on gender and age.

**Results:**

A total of 1,418 participants were included, with 43.1% showing cognitive impairment. Multivariate analysis revealed that the risk of cognitive impairment decreased by 0.2% for each unit increase in UA level and was reduced by 33% in the third quartile of UA levels compared with the lowest quartile (OR = 0.67, 95% CI: 0.49, 0.92, *p* = 0.014). MMSE scores increased by 0.01 (*β* = 0.01, 95% CI: 0.002, 0.01, *p* = 0.006) for each unit increase in UA level. Subgroup analysis showed significant protective associations in men and participants aged 60–69 years. However, no such relationship was found in women or individuals aged ≥70 years.

**Conclusion:**

This study highlights the cognitive protective effect of UA in low-income rural Chinese populations aged ≥60 years without hyperuricemia, particularly in men and those aged 60–69 years. These findings underscore the importance of targeted interventions and health education programs to prevent cognitive decline in this vulnerable population.

## 1 Introduction

The Global Burden of Disease (GBD) study estimates that over 56.9 million people worldwide are affected by Alzheimer’s disease and other dementias, resulting in 36.3 million disability-adjusted life years (DALYs) lost (1). Between 1990 and 2016, the prevalence of dementia increased by 117%, and dementia-related deaths rose by 148% (2). Mild cognitive impairment (MCI), an intermediary stage between normal aging and dementia, is widely recognized as a precursor to dementia (3). In China, the prevalence rates of dementia and MCI are 6.0 and 15.5%, respectively, which has imposed a tremendous burden on the whole society, especially in rural areas (4, 5). Therefore, early identification and prevention of risk factors for cognitive impairment are of utmost importance.

Uric acid (UA), the final product of purine nucleotide metabolism, has been suggested to have a neuroprotective role through its free radical scavenging activity. However, UA-induced endothelial dysfunction and inflammation may also negatively impact cognitive function (6–9). The relationship between UA levels and cognitive function remains controversial, with conflicting results reported across various studies (10–15). Particularly in non-hyperuricemic populations. Some studies suggest that UA may have a protective effect on cognitive function in individuals without hyperuricemia. One study based on the China Health and Retirement Longitudinal Study (CHARLS) found that lower baseline UA levels were associated with poorer cognitive performance in individuals without hyperuricemia (16). Another study on Chinese adults demonstrated that higher baseline UA levels were linked to better baseline cognition and less subsequent cognitive decline in those without hyperuricemia (17). Additionally, research in populations with abnormal blood pressure similarly found that moderately elevated UA levels within the normal range were associated with improved cognitive function (18). However, some studies have shown that UA is associated with poorer cognitive performance in individuals without hyperuricemia. A 9-year prospective study also indicated that higher UA levels were related to a reduced risk of MCI in elderly Chinese individuals without hyperuricemia (19). A Kailuan Study-based research revealed that in analyses of non-hyperuricemia participants, elevated serum UA levels were associated with reduced white matter volume, lower fractional anisotropy values, and poorer cognitive performance (20). However, the relationship between UA and cognitive function is controversial. Previous studies have mostly focused on urban areas, and there is a lack of research on low-income rural residents. It is currently unclear what the relationship between UA levels and cognitive function in low-income rural residents without hyperuricemia.

In China, the health issues of low-income rural populations are particularly prominent, as this group faces unique health challenges and socioeconomic barriers. Compared to urban residents, rural residents often encounter more difficulties in accessing medical services and health information, which may lead to a higher risk of cognitive impairment. From an epidemiological perspective, residents in rural areas may exhibit different disease epidemiological characteristics, which could be related to the occurrence and development of cognitive impairment. Meanwhile, from an economic standpoint, low income may limit residents’ access to health services, affecting their ability to obtain preventive health measures, thereby potentially increasing the risk of cognitive impairment.

Thus, this study conducted a cross-sectional study based on low-income rural residents aged ≥60 years in Tianjin, China, aiming to explore the correlation between UA levels and cognitive function in low-income rural residents without hyperuricemia, and further explore its association in rural low-income people without hyperuricemia with different characteristics through subgroup analysis. We selected this specific population based on our understanding of the current health inequalities in rural China and the urgent need to improve the health status of this group. We hope that through this study, we can provide a scientific basis for developing more targeted public health interventions, thereby enhancing the cognitive health and quality of life of elderly people in rural areas.

## 2 Method

### 2.1 Study population

This cross-sectional study was conducted in 2012, 2019 and 2020, involving a low-income rural population from Tianjin, China. The subjects of this study were from a township in rural Tianjin, China. Approximately 95% of them were low-income households, with an average annual income per capita below $100 in 1991 and below $3,000 in 2019 (21). Participants aged 60 years and older without visual or auditory impairments were invited to participate. Exclusion criteria included the presence of hyperuricemia or incomplete Mini-Mental State Examination (MMSE) data (22). The study adhered to the principles of the Declaration of Helsinki and was approved by the Ethics Committee of Tianjin Medical University General Hospital (IRB-2018-100-01). All participants provided written informed consent.

### 2.2 Data collection

Data were collected through face-to-face interviews conducted by trained researchers. Sociodemographic and clinical information was obtained, including participants’ name, gender, age, education level, lifestyle, history of diabetes and hypertension, fasting blood glucose (FBG), total cholesterol (TC), triglycerides (TG), UA. UA levels were categorized into quartiles. Physical examinations were performed to measure height and weight, with participants wearing light clothing during the assessment. All measurements were recorded by the same researcher to minimize systematic error. Hypertension was defined as a systolic blood pressure ≥140 mmHg or a diastolic blood pressure ≥90 mmHg on three separate occasions or the use of antihypertensive medications, or a self-reported history of hypertension. Diabetes was defined as an FBG level ≥7.0 mmol/L, the use of diabetes medications, or a self-reported history of diabetes.

Smoking status was defined as individuals who smoked at least one cigarette per day for over one year; if the ethanol intake reached or exceeded 45 grams per day in the previous year, it was considered positive alcohol consumption. Blood samples were collected in the morning after participants fasted for at least 12 h.

Cognitive function was assessed using the MMSE, a widely used tool for screening cognitive impairment. The MMSE includes items on memory, orientation, language, attention, and calculation, with a total score of 30. The criteria for diagnosing cognitive impairment are consistent with the previous standards established by Liu et al. for China Health and Retirement Longitudinal Study (CHARLS) (23). Cognitive impairment was defined based on education level: an MMSE score below 18 for illiterate individuals, below 21 for those with 6 years or less of education, and below 25 for those with more than 6 years of education. The clinical information of the subjects and the MMSE assessment were collected in the same year. Both assessments were conducted within the same timeframe to ensure data consistency and accuracy.

### 2.3 Statistical analysis

Continuous variables were expressed as mean and standard deviation (SD) and compared using a t-test. Categorical variables are expressed as frequency and percentage and compared using chi-square tests. The factors which were related to cognitive impairment and MMSE score (*p* < 0.05) in univariate analysis were included in the respective multiple factor analysis models. Binary logistic regression analysis was used to examine the relationship between UA and cognitive impairment, and a linear regression model was used to verify the linear relationship between UA and MMSE score, adjusting for the potential confounders identified in univariate analysis (*p* < 0.05). The association between UA levels and cognitive impairment and MMSE scores was expressed as adjusted odds ratios (ORs) with 95% confidence intervals (CIs) or *β* values. Subgroup analyses were performed by sex (male, female) and age group (60–69 years, ≥ 70 years) to further explore their relevance. To further investigate the nonlinear relationship between UA levels, cognitive impairment risk, and MMSE score, we plotted nonlinear cubic spline diagrams to clarify the association between UA and outcomes.

In this study, to explore the relationship between UA levels and cognitive function, we categorized the participants’ serum UA levels into four quartiles (Q1, Q2, Q3, Q4). This grouping was based on the percentile distribution of UA levels, where Q1 represents the lowest 25% of participants by UA level, Q2 represents the next 25%, Q3 denotes the following 25%, and Q4 indicates the highest 25% of participants by UA level. The specific criteria for UA grouping in this study are as follows: The range of UA in this study was 32.28–420.00 μmol/L, with a range of 387.72 μmol/L. Q1 includes UA levels at or below the 25th percentile of 228.32 μmol/L; Q2 covers the range from the 25th percentile of 228.32 μmol/L to the median of 272.00 μmol/L; Q3 comprises UA levels from the median of 272.00 μmol/L to the 75th percentile of 320.00 μmol/L; Q4 includes UA levels above the 75th percentile of 320.00 μmol/L. By using this method of grouping, we aimed to meticulously analyze the potential non-linear relationship between UA levels and cognitive function, thereby enhancing the interpretability and robustness of the statistical analysis.

In this study, age was stratified into two groups—60-69 years and ≥70 years—based on age-related physiological and cognitive changes. The 60–69 age group represents early aging, while the ≥70 age group represents late aging. Individuals in these two age groups may experience varying rates of physiological decline and different risks of neurodegenerative diseases. The aim is to explore the potential differences in how UA levels affect cognitive function across different age groups.

All statistical analyses were performed using SPSS version 27.0. For primary analyze, Bonferroni-adjusted thresholds were applied (*α* = 0.05/m, where m is the number of tests). R version 4.4.0 is used for drawing.

## 3 Result

### 3.1 Demographic characteristics

**Figure 1:**
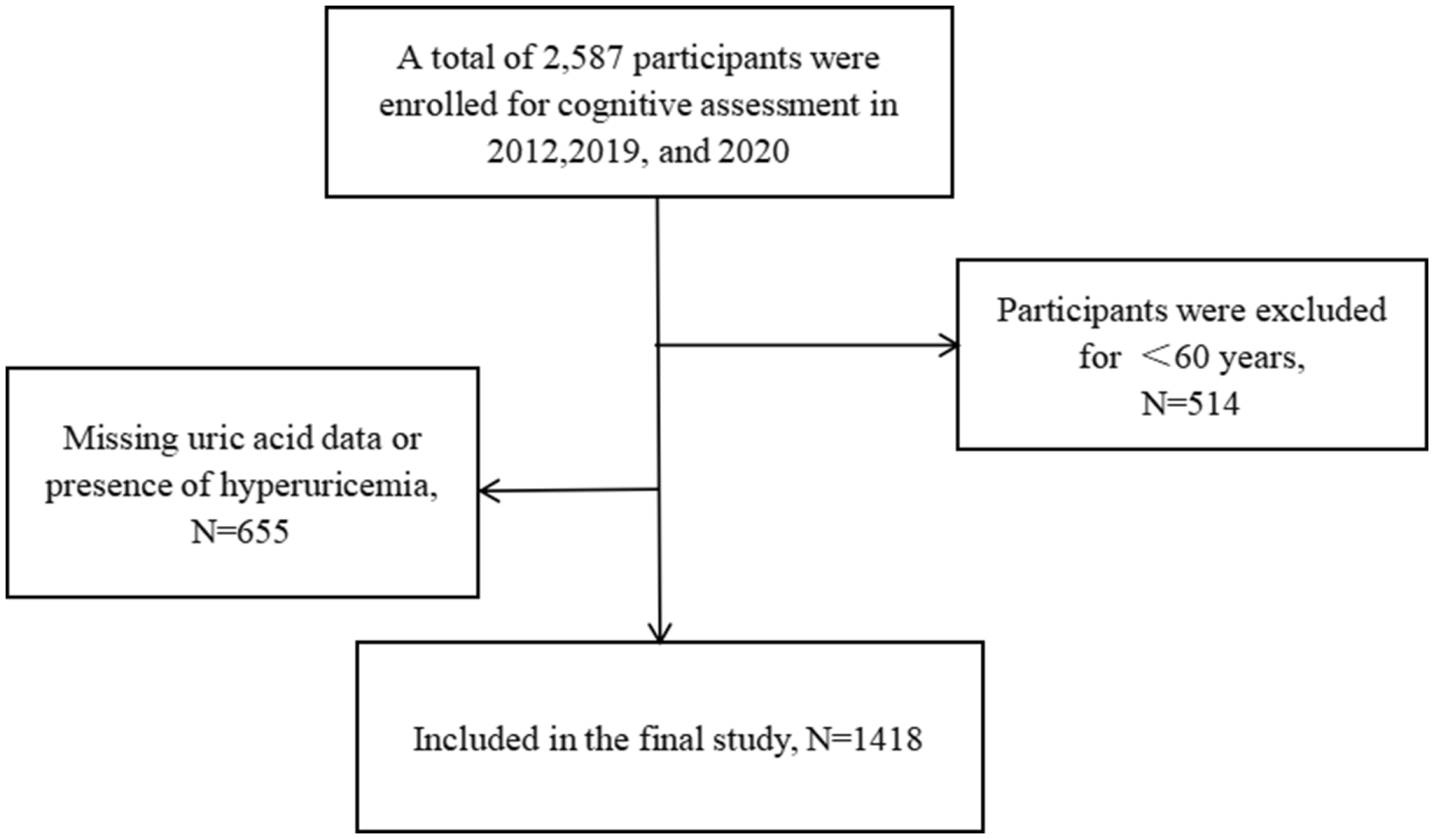
Flowchart of the screening process.

A total of 1,418 participants were included in this study, of whom 42.2% (*n* = 599) were males and 57.8% (*n* = 819) were females (Figure 1). The mean age of the participants was 66.37 ± 5.34 years. The prevalence of cognitive impairment in the study population was 43.1% (*n* = 607). Other characteristics of the participants are detailed in Table 1.

**Table 1:** Characteristics of participants.

Characteristics	Men	Women	Total
Case, *n* (%)	599 (42.2)	819 (57.8)	1,418 (100)
Age, years, means ± SD	66.56 ± 5.37	66.17 ± 5.31	66.37 ± 5.34
Age groups, *n* (%)			
60–69 years	455 (76.0)	640 (78.1)	1,095 (77.2)
≥70 years	144 (24.0)	179 (21.9)	323 (22.8)
Education years, means ± SD	6.09 ± 2.91	3.06 ± 2.30	4.33 ± 3.48
Education groups, *n* (%)			
0 years	33 (5.6)	325 (39.8)	358 (25.4)
1–6 years	335 (56.6)	375 (45.9)	710 (50.4)
>6 years	224 (37.8)	117 (14.3)	341 (24.2)
Hypertension, *n* (%)	378 (63.1)	604 (73.7)	982 (69.3)
Diabetes, *n* (%)	86 (14.4)	207 (25.3)	293 (20.7)
BMI, Kg/m^2^, means ± SD	24.36 ± 3.34	25.42 ± 3.63	24.97 ± 3.55
BMI groups, *n* (%)			
Under weight	22 (3.7)	16 (2.0)	38 (2.7)
Normal	262 (44.0)	275 (33.7)	537 (38.0)
Overweight	228 (38.3)	349 (42.8)	577 (40.9)
Obesity	84 (14.1)	176 (21.6)	260 (18.4)
Smoking, *n* (%)			
Never smoking	104 (17.4)	755 (93.0)	859 (60.9)
Current smoking	280 (46.8)	18 (2.2)	298 (21.1)
Ever smoking	214 (35.8)	39 (4.8)	253 (17.9)
Alcohol consumption, *n* (%)			
Never drinking	160 (26.8)	776 (95.2)	936 (66.2)
Current drinking	309 (51.7)	19 (2.3)	328 (23.2)
Ever drinking	129 (21.6)	20 (2.5)	149 (10.5)
Uric acid, means ± SD	295.78 ± 63.94	259.17 ± 59.96	274.64 ± 64.25
Uric acid groups, *n* (%)			
Q1	92 (15.4)	262 (32.0)	354 (25.0)
Q2	130 (21.7)	226 (27.6)	356 (25.1)
Q3	161 (26.9)	195 (23.8)	356 (25.1)
Q4	216 (36.1)	136 (16.6)	352 (24.8)
FBG, mmol/L, means ± SD	5.61 ± 1.41	5.88 ± 1.69	5.77 ± 1.58
TG, mmol/L, means ± SD	1.48 ± 1.24	1.75 ± 1.04	1.63 ± 1.13
TC, mmol/L, means ± SD	4.43 ± 1.00	4.83 ± 1.09	4.66 ± 1.07
Cognitive impairment, *n* (%)	174 (29.4)	433 (53.0)	607 (43.1)

SD, standard deviation; BMI, body mass index; FBG, fasting blood glucose; TG, triglycerides; TC, total cholesterol; Q1-Q4, uric acid quartile groups.

### 3.2 Univariate analysis of factors influencing cognitive impairment

Table 2 shows the results of univariate analysis, and there were differences in gender, age, age group, smoking history, alcohol consumption history, UA, and UA group levels between the cognitive normal group and the cognitive impairment group (*p* < 0.05). Specifically, female, elderly, non-smoking, non-drinking, and low UA levels were associated with cognitive impairment (all *p* < 0.05). Following Bonferroni’s correction (*p* < 0.004 threshold), these factors remained statistically significant (*p* < 0.004).

**Table 2:** Results of a univariate analysis of clinical characteristics and cognitive impairment.

Characteristics	Cognitive normal	Cognitive impairment	*p*
Men, *n* (%)	418 (52.1)	174 (28.7)	<0.001
Age, means ± SD	65.50 ± 4.45	67.52 ± 6.15	<0.001
Age groups, *n* (%)			<0.001
60–69 years	674 (84.0)	414 (68.2)	
≥70 years	128 (16.0)	193 (31.8)	
Hypertension, *n* (%)	544 (67.8)	436 (71.8)	0.106
Diabetes, *n* (%)	156 (19.5)	136 (22.4)	0.176
BMI, Kg/m^2^, means ± SD	25.01 ± 3.40	24.90 ± 3.72	0.575
BMI groups, *n* (%)			0.948
Under weight	21 (2.6)	17 (2.8)	
Normal	300 (37.6)	233 (38.5)	
Overweight	333 (41.7)	243 (40.2)	
Obesity	144 (18.0)	112 (18.5)	
Smoking, *n* (%)			<0.001
Never smoking	415 (51.9)	437 (72.6)	
Current smoking	211 (26.4)	85 (14.1)	
Ever smoking	173 (21.7)	80 (13.3)	
Alcohol consumption, *n* (%)			<0.001
Never drinking	464 (58.0)	467 (77.2)	
Current drinking	240 (30.0)	86 (14.2)	
Ever drinking	96 (12.0)	52 (8.6)	
Uric acid, means ± SD	280.77 ± 64.02	266.34 ± 63.99	<0.001
Uric acid groups, *n* (%)			<0.001
Q1	173 (21.6)	180 (29.7)	
Q2	195 (24.3)	160 (26.4)	
Q3	217 (27.1)	133 (21.9)	
Q4	217 (27.1)	134 (22.1)	
FBG, mmol/L	5.69 ± 1.50	5.86 ± 1.66	0.054
TG, mmol/L	1.63 ± 1.22	1.64 ± 1.02	0.950
TC, mmol/L	4.62 ± 1.07	4.71 ± 1.07	0.125

SD, standard deviation; BMI, body mass index; FBG, fasting blood glucose; TG, triglycerides; TC, total cholesterol; Q1-Q4, uric acid quartile groups.

### 3.3 Multivariate analysis of UA and cognitive impairment

After adjusting for gender, age group, smoking history, and alcohol consumption history, the results of multivariate analysis showed that the risk of cognitive impairment decreased by 0.2% for each unit increase in UA level (OR = 0.998, 95%CI: 0.996, 1.000; *p* = 0.025), and the risk of cognitive impairment in the third group (Q3) decreased by 33% compared with the lowest UA level group (Q1) (OR = 0.67, 95%CI: 0.49, 0.92; *p* = 0.014). In the UA model, the risk of cognitive impairment was increased by 89% in women compared with men (OR = 1.89, 95%CI: 1.27, 2.80; *p* = 0.002). Compared with the 60–69 age group, the probability of developing cognitive impairment increased by 170% ≥ the 70-year-old age group (OR = 2.70, 95%CI: 2.06, 3.53; *p* < 0.001). In the UA grouping model, the risk of cognitive impairment was increased by 91% in women compared with men (OR = 1.91, 95%CI: 1.29, 2.84; *p* = 0.001). Compared with the 60–69 age group, the probability of developing cognitive impairment increased by 168% ≥ the 70-year-old age group (OR = 2.68, 95% CI: 2.04, 3.51; *p* < 0.001) (Table 3).

**Table 3:** Results of multivariate analysis between uric acid and cognition impairment.

Characteristics	References	OR (95%CI)	*p*
Model 1			
Uric acid		0.998 (0.996, 1.000)	0.025
Women	Men	1.89 (1.27, 2.80)	0.002
Age groups	60–69 years		
≥70 years		2.70 (2.06, 3.53)	<0.001
Smoking	Never smoking		
Current smoking		0.79 (0.52, 1.21)	0.282
Ever smoking		0.71 (0.47, 1.08)	0.108
Alcohol consumption	Never drinking		
Current drinking		0.75 (0.50, 1.11)	0.144
Ever drinking		0.98 (0.62, 1.53)	0.921
Model 2			
Uric acid groups	Q1		
Q2		0.85 (0.63, 1.16)	0.310
Q3		0.67 (0.49, 0.92)	0.014
Q4		0.76 (0.55, 1.05)	0.095
Women	Men	1.91 (1.29, 2.84)	0.001
Age groups	60–69 years		
≥70 years		2.68 (2.04, 3.51)	<0.001
Smoking	Never smoking		
Current smoking		0.79 (0.51, 1.20)	0.267
Ever smoking		0.71 (0.47, 1.07)	0.105
Alcohol consumption	Never drinking		
Current drinking		0.75 (0.50, 1.11)	0.143
Ever drinking		0.98 (0.63, 1.53)	0.927

OR, Odds Ratio; 95%CI, 95% confidence interval; Q1-Q4, uric acid quartile groups.

### 3.4 Univariate analysis of factors influencing MMSE scores

Univariate analysis showed that gender, age, age group, smoking history, alcohol consumption history, hypertension, diabetes, UA level, high blood glucose level, and high TC level were associated with lower MMSE score (all *p* < 0.05) (Table 4).

**Table 4:** Results of a univariate analysis of clinical characteristics and MMSE scores.

Characteristics	Reference	*β* (95%CI)	*p*
Women	Men	−3.32 (−3.83, −2.81)	<0.001
Age		−0.26 (−0.31, −0.21)	<0.001
Age groups	60–69 years	−2.90 (−3.52, −2.28)	<0.001
BMI		0.04 (−0.04, 0.12)	0.299
BMI groups	Under weight	0.11 (−0.23, 0.45)	0.534
Smoking	Never smoking	1.52 (1.19, 1.85)	<0.001
Alcohol consumption	Never drinking	1.58 (1.19, 1.96)	<0.001
Hypertension		−0.60 (−1.18, −0.02)	0.042
Diabetes		−0.84 (−1.50, −0.18)	0.012
Uric acid		0.01 (0.01, 0.02)	<0.001
Uric acid groups		0.57 (0.33, 0.80)	<0.001
FBG		−0.25 (−0.42, −0.09)	0.003
TG		−0.06 (−0.30, 0.18)	0.620
TC		−0.28 (−0.54, −0.03)	0.030

BMI, body mass index; FBG, fasting blood glucose; TG, triglycerides; TC, total cholesterol; 95%CI, 95% confidence interval.

Following Bonferroni’s correction (*p* < 0.004 threshold), gender, age, age group, smoking history, alcohol consumption history, UA level, high blood glucose level were associated with lower MMSE score (*p* < 0.004).

### 3.5 Multivariate analysis of UA and MMSE scores

Table 5 shows that after adjusting for gender, age group, smoking history, alcohol consumption history, hypertension, diabetes, and TC, (without Bonferroni’s correction) multivariate analysis showed that the MMSE score increased by 0.01 (*β* = 0.01, 95%CI: 0.002, 0.01; *p* = 0.006) for each unit of UA level, and the MMSE score increased by 0.33 (*β* = 0.33, 95%CI: 0.10, 0.56; *p* = 0.006) for each additional UA group level. In the UA model, compared with men, the MMSE score of women decreased by 2.96 (*β* = −2.96, 95%CI: −3.73, −2.19; *p* < 0.001), and compared with the 60–69 age group, the MMSE score of ≥ 70-year-old group decreased by 2.94 (*β* = −2.94, 95%CI: −3.53, −2.34; *p* < 0.001). In the UA grouping model, the MMSE score of women decreased by 2.98 (*β* = −2.98, 95%CI: −3.75, −2.12; *p* < 0.001) compared with men, and the MMSE score decreased by 2.94 (*β* = −2.94, 95%CI: −3.53, − 2.34; *p* < 0.001) in the 70-year-old age group compared with the 60–69 age group.

**Table 5:** Results of multivariate analysis between uric acid and MMSE scores.

Characteristics	Reference	*β* (95%CI)	*p*
Model 1			
Uric acid		0.01 (0.002, 0.01)	0.006
Women	Men	−2.96 (−3.73, −2.19)	<0.001
Age groups	60–69 years	−2.94 (−3.53, −2.34)	<0.001
Smoking	Never smoking	0.21 (−0.23, 0.66)	0.349
Alcohol consumption	Never drinking	−0.06 (−0.55, 0.43)	0.808
Hypertension		−0.36 (−0.92, 0.20)	0.210
Diabetes		−0.24 (−0.87, 0.39)	0.453
TC		0.06 (−0.18, 0.30)	0.642
Model 2			
Uric acid groups	Q1	0.33 (0.10, 0.56)	0.006
Women	Men	−2.98 (−3.75, −2.12)	<0.001
Age groups	60–69 years	−2.94 (−3.53, −2.34)	<0.001
Smoking	Never smoking	0.21 (−0.24, 0.66)	0.356
Alcohol consumption	Never drinking	−0.06 (−0.55, 0.43)	0.802
Hypertension		−0.36 (−0.92, 0.20)	0.206
Diabetes		−0.24 (−0.86, 0.39)	0.461
TC		0.05 (−0.19, 0.29)	0.665

TC, total cholesterol; 95%CI, 95% confidence interval; Q1-Q4, uric acid quartile groups.

After applying Bonferroni’s correction, the multivariate analysis results showed that, for every 1-unit increase in UA level, the MMSE score increased by 0.01 (*β* = 0.01, 95% CI: 0.001, 0.01; *p* = 0.010); for every 1-unit increase in UA group level, the MMSE score increased by 0.30 (*β* = 0.30, 95% CI: 0.07, 0.53; *p* = 0.010). In the UA model, women had a 2.91-point lower MMSE score compared to men (*β* = −2.91, 95% CI: −3.67, −2.16; *p* < 0.001); and the MMSE score was 2.92 points lower in the ≥70-year-old group compared to the 60-69-year-old group (*β* = −2.92, 95% CI: −3.51, −2.33; *p* < 0.001). In the UA grouping model, women had a 2.93-point lower MMSE score than men (*β* = −2.93, 95% CI: −3.68, −2.18; *p* < 0.001); and the MMSE score in the 70-year-old group was 2.92 points lower than that in the 60-69-year-old group (*β* = −2.92, 95% CI: −3.51, −2.33; *p* < 0.001) (Table 6).

**Table 6:** Results of multivariate analysis between uric acid and MMSE scores after Bonferroni’s correction.

Characteristics	Reference	*β* (95%CI)	*p*
Model 1			
Uric acid		0.01 (0.001, 0.01)	0.010
Women	Men	−2.91 (−3.67, −2.16)	<0.001
Age groups	60–69 years	−2.92 (−3.51, −2.33)	<0.001
Smoking	Never smoking	0.26 (−0.18, 0.71)	0.239
Alcohol consumption	Never drinking	−0.03 (−0.52, 0.45)	0.897
FBG		−0.11 (−0.26, 0.05)	0.177
Model 2			
Uric acid groups	Q1	0.30 (0.07, 0.53)	0.010
Women	Men	−2.93 (−3.68, −2.18)	<0.001
Age groups	60–69 years	−2.92 (−3.51, −2.33)	<0.001
Smoking	Never smoking	0.26 (−0.18, 0.70)	0.243
Alcohol consumption	Never drinking	−0.03 (−0.52, 0.45)	0.890
FBG		−0.11 (−0.27, 0.05)	0.174

95%CI, 95% confidence interval; Q1-Q4, uric acid quartile groups.

### 3.6 Multivariate analysis of UA and cognition impairment/MMSE in subgroups

**Figure 2:**
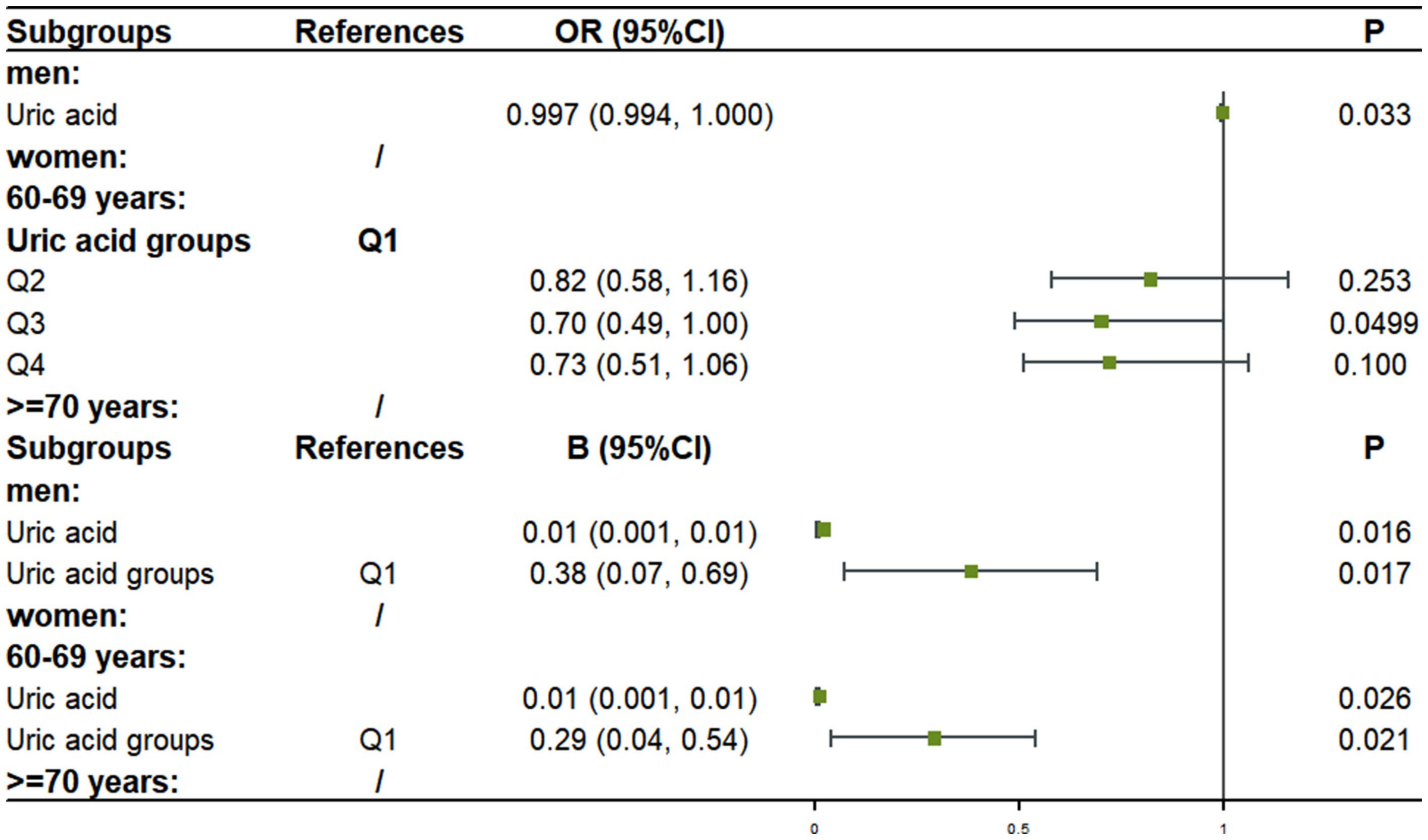
Subgroup results of multivariate analysis of uric acid and cognitive function/MMSE.

Subgroup analysis according to age and gender showed that in the male subgroup without hyperuricemia, For each unit increase in UA level, the risk of cognitive impairment decreased by 0.3% (OR = 0.997, 95%CI: 0.994, 1.000; *p* = 0.033), and MMSE score increased by 0.01 (*β* = 0.01, 95%CI: 0.001, 0.01; *p* = 0.016), MMSE score increased by 0.38 (*β* = 0.38, 95%CI: 0.07, 0.69; *p* = 0.017) for each additional UA group. In the 60–69 years subgroup, the risk of developing cognitive impairment in Q3 decreased by 30% compared with Q1 (OR = 0.70, 95% CI: 0.49, 1.00; *p* = 0.0499), MMSE score increased by 0.01 (*β* = 0.01, 95% CI: 0.001, 0.01; *p* = 0.026) for each unit of UA increase, and MMSE score increased by 0.29 (*β* = 0.29, 95% CI: 0.04, 0.54; *p* = 0.021) for each additional UA group, there was no statistically significant association between UA level and cognitive function or MMSE score in women and the subgroup of ≥ 70 years (all *p* > 0.05) (Figure 2).

### 3.7 Nonlinear association analysis between UA level and cognitive impairment/MMSE

**Figure 3:**
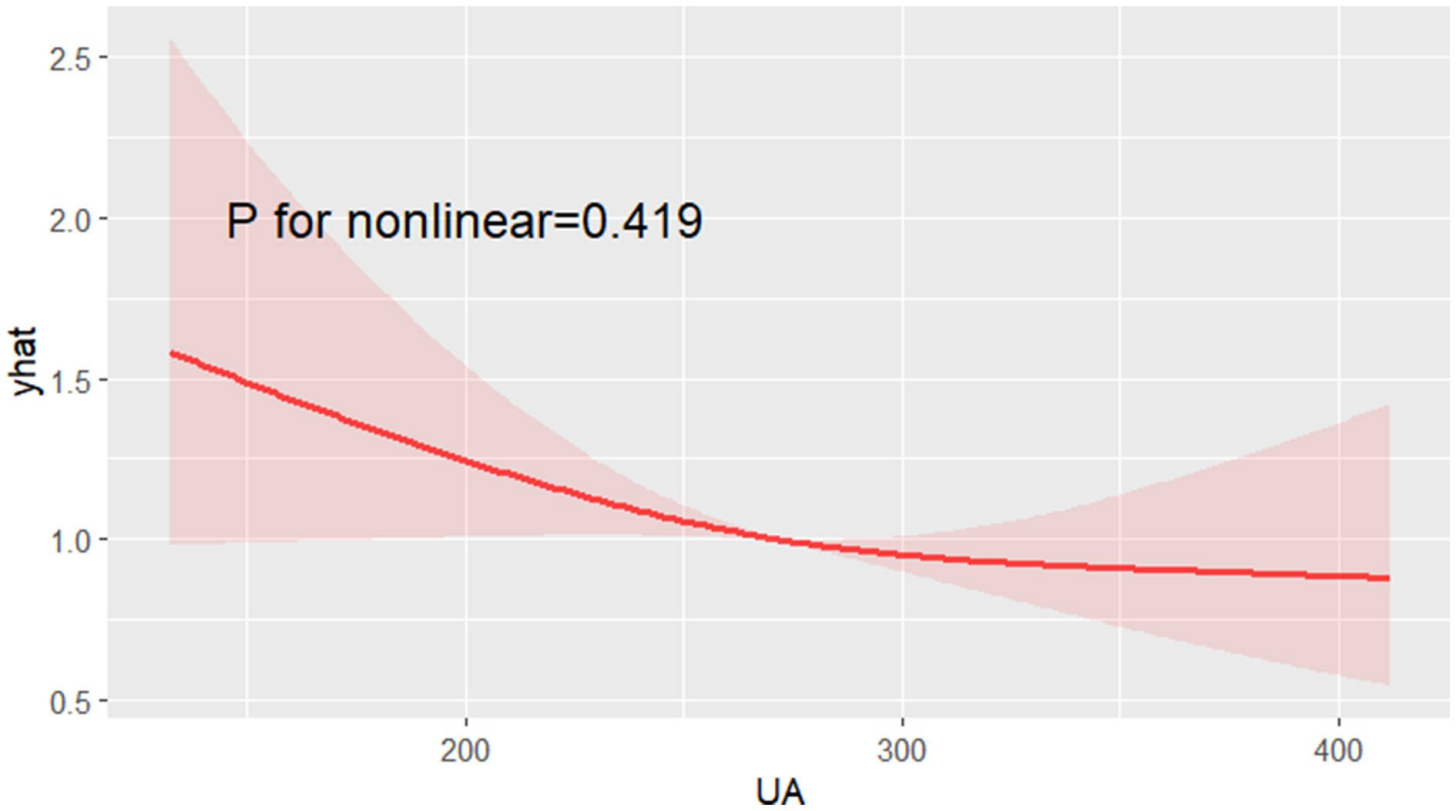
Nonlinear analysis results of uric acid and cognitive function.

**Figure 4:**
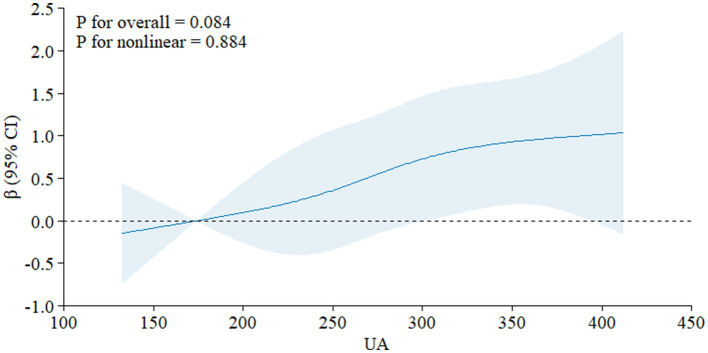
Nonlinear analysis results of uric acid and MMSE.

Analysis using restricted cubic spline graphs showed no significant nonlinear association between UA levels and the risk of cognitive impairment (Figure 3) or MMSE scores (Figure 4) (all *p* > 0.05).

## 4 Discussion

This study aimed to investigate the association between UA levels and cognitive impairment among residents aged 60 and above in low-income rural areas of Tianjin, China, without hyperuricemia. The findings indicate a significant association between UA levels and cognitive function, with individuals having higher UA levels exhibiting a relatively lower risk of cognitive impairment. Moreover, the protective effect of UA on cognitive function is particularly evident in males and those aged 60 to 69 years, while this association is not apparent in females and those aged 70 years and above. Notably, in the univariate analysis, cognitive impairment and MMSE scores showed distinct sets of covariates. Mechanistically speaking, the former may reflect crossing specific education-level MMSE thresholds, thereby mirroring overall structural damage or substantial synaptic loss, whereas the latter is more sensitive to subtle changes across the entire cognitive spectrum. This discovery underscores the potential protective role of UA in cognitive health and points to possible differential impacts across genders and age groups.

Although numerous studies have investigated the association between UA levels and cognitive dysfunction in non-hyperuricemic populations, current research findings remain highly inconsistent. Studies on rural areas have not been reported. Some studies suggest that UA has a protective effect on cognitive function. A study from the CHARLS showed that low plasma levels of UA adversely affected cognitive function in Chinese adults without hyperuricemia (16). Another study of Chinese adults without hyperuricemia showed that higher baseline serum UA levels were associated with better baseline cognition and less subsequent cognitive decline, with depression playing a mediating role (17). Jiang et al. also showed that in non-hyperuricemia populations, elevated UA levels were associated with better cognitive function, particularly in those with lower levels of inflammation (24). However, analysis based on the Kailuan study demonstrated that elevated serum UA levels were associated with reduced white matter volume, decreased anisotropy fraction, and cognitive decline in non-hyperuricemic subjects (20). The results of this study in low-income rural populations show that UA plays a protective role in cognitive function in low-income rural populations without hyperuricemia, especially in men and 60–69 years adults. The protective effect of UA on cognitive function may be achieved through several pathways. UA, as an antioxidant, can play a role in cognitive protection by inhibiting the reaction process of peroxynitrite to reduce oxidative damage in the body (25–27). In addition, UA transporter gene markers have also been observed to be associated with individual cognitive ability (28). However, this protective effect appears to be present only in people without hyperuricemia and not in hyperuricemia (29). The study by Zhang et al. demonstrated that early UA levels were negatively correlated with diffusion tensor imaging (DTI) metrics in the brain, while the variability of UA levels exhibited a nonlinear U-shaped relationship with DTI parameters. These findings suggest that maintaining moderate and stable UA levels may have protective effects on cognitive health (30). More large-scale prospective experiments are needed in the future to verify our experimental conclusions.

Previous studies have generally shown that both female sex and advanced age are risk factors for cognitive impairment. Our study also confirms that both women and older are at increased risk of cognitive impairment. Women are at higher risk of dementia and have more significant cognitive deterioration than men (31–33). Although many studies have verified this, the mechanisms underlying it are unclear and may be related to a variety of pathophysiological processes, such as differences in sex hormones, differences in genetic risk, differences in brain anatomy, and differences in metabolism between men and women (31, 34, 35). Older age is a common risk factor for cognitive impairment, and with increasing age, oxidative stress, changes in cerebral blood flow, and changes in the endocrine system can lead to cognitive decline (36–39).

Previous studies have shown that the effects of UA on cognition are more sensitive in men, and the protective effect of serum UA on cognition appears to be present only in men but not in women (15, 39). Our findings also suggest that UA’s protective effect on cognitive function is only present in the male subgroup, but not in the female subgroup. This may be due to the fact that elevated UA levels have different effects on spontaneous brain activity and cognitive function in men and women, with men being more susceptible to changes (40).

Previous studies have shown that UA’s protective effect on cognitive function is age-related. The experimental results of Huang et al. showed that stronger UA was observed in non-hyperuricemia people aged ≥ 60 years at baseline (16). In another study of patients with cardiovascular disease, the effect of UA on cognitive impairment was more pronounced in patients > 65 years of age (41). A prospective study by Chen et al. found that the protective effect of high UA levels on cognitive function was more pronounced in younger older adults (19). This is consistent with our findings, which also show that the protective effect of UA on cognition in rural low-income people without hyperuricemia is more pronounced in the subgroup of 60–69 year olds rather than the ≥ 70-year-old subgroup. This may be because younger older people age at a slower rate and are more sensitive to the protective effects of UA (42). However, there is little evidence from current studies, and more experiments should be conducted in the future to verify our conclusions.

Our study has several limitations. First, as a cross-sectional study, it cannot establish a causal relationship between UA levels and cognitive function in rural, low-income populations without hyperuricemia. Future prospective cohort studies are needed to explore the causal links between UA and cognitive impairment. Second, our study population was limited to rural areas in Tianjin, which may reduce the generalizability of our findings to other populations, such as urban residents or those from different socioeconomic backgrounds or geographic regions. Future research should include diverse populations from various regions and socioeconomic statuses to improve the generalizability of the findings. Third, using the MMSE as the sole tool for assessing cognitive function may be insufficient. The MMSE may not capture all dimensions of cognitive impairment, potentially leading to underestimation or misclassification of cognitive status. Future studies should employ a comprehensive range of cognitive assessments, including tests targeting different cognitive domains such as memory, executive function, and processing speed, to provide a more detailed and accurate evaluation of cognitive impairment. Fourth, potential confounding variables, such as dietary habits, physical activity levels, and genetic factors, were not controlled for in this study. These factors could simultaneously influence both UA levels and cognitive function, thereby affecting the observed associations. Future research should collect and control for a broader range of confounding variables to more accurately isolate the effect of UA on cognitive impairment. Including comprehensive lifestyle and genetic data will strengthen the robustness of the study’s conclusions. Additionally, some data, such as smoking and drinking history, were self-reported. Self-reported data are subject to recall bias and social desirability bias, which may result in inaccurate reporting and affect the study results. Utilizing objective measurement methods and validating self-reported data through medical records or biochemical markers will enhance data accuracy and reliability.

In conclusion, this study investigated the relationship between UA levels and cognitive impairment in rural low-income populations aged ≥60 years without hyperuricemia in rural low-income populations in Tianjin, China. The findings suggest that UA can reduce the risk of cognitive impairment and increase MMSE scores. The results of subgroup analysis showed that this association also existed in the male and 60–69 years old subgroup, but not in the female and ≥70 subgroup populations, highlighting the important role of UA in the cognitive health of rural low-income populations without hyperuricemia, and the importance of individualized and targeted health strategies for different populations. By linking the cognitive protective effects of UA in special populations, healthcare providers and policymakers can develop more effective interventions to improve cognitive function and overall well-being in rural, low-income communities.

## Data Availability

The original contributions presented in the study are included in the article/supplementary material, further inquiries can be directed to the corresponding author.
